# Exploring the Remarkable Chemotherapeutic Potential of Polyphenolic Antioxidants in Battling Various Forms of Cancer

**DOI:** 10.3390/molecules28083475

**Published:** 2023-04-14

**Authors:** Mohammad Imran, Areeba Insaf, Nazeer Hasan, Vrushabh V. Sugandhi, Deumaya Shrestha, Keshav Raj Paudel, Saurav Kumar Jha, Philip M. Hansbro, Kamal Dua, Hari Prasad Devkota, Yousuf Mohammed

**Affiliations:** 1 Therapeutics Research Group, Frazer Institute, Faculty of Medicine, The University of Queensland, Brisbane, QLD 4102, Australia; mohammad.imran@uq.edu.au; 2 Department of Pharmacognosy and Phytochemistry, School of Pharmaceutical Education and Research, Jamia Hamdard, New Delhi 110062, India; 3 Department of Pharmaceutics, School of Pharmaceutical Education and Research, Jamia Hamdard, New Delhi 110062, India; 4 Department of Pharmaceutics, Y.B. Chavan College of Pharmacy, Aurangabad 431001, India; 5 Department of Bioscience, Mokp o National University, Muna 58554, Republic of Korea; 6 Centre of Inflammation, School of Life Sciences, Faculty of Science, Centenary Institute and University of Technology Sydney, Sydney, NSW 2007, Australia; 7 Department of Biomedicine, Health & Life Convergence Sciences, Mokpo National University, Muna 58554, Republic of Korea; 8 Discipline of Pharmacy, Graduate School of Health, University of Technology Sydney, Sydney, NSW 2007, Australia; 9 Faculty of Health, Australian Research Centre in Complementary and Integrative Medicine, University of Technology Sydney, Ultimo, NSW 2007, Australia; 10 Graduate School of Pharmaceutical Sciences, Kumamoto University, 5-1 Oe-honmachi, Kumamoto 862-0973, Japan; 11 Pharmacy Program, Gandaki University, Pokhara 33700, Nepal; 12 School of Pharmacy, The University of Queensland, Brisbane, QLD 4102, Australia

**Keywords:** flavonoids, antioxidant, anticancer, nanotechnology, combination therapy

## Abstract

Plant-derived compounds, specifically antioxidants, have played an important role in scavenging the free radicals present under diseased conditions. The persistent generation of free radicals in the body leads to inflammation and can result in even more severe diseases such as cancer. Notably, the antioxidant potential of various plant-derived compounds prevents and deregulates the formation of radicals by initiating their decomposition. There is a vast literature demonstrating antioxidant compounds’ anti-inflammatory, anti-diabetic, and anti-cancer potential. This review describes the molecular mechanism of various flavonoids, such as quercetin, kaempferol, naringenin, epicatechin, and epicatechin gallate, against different cancers. Additionally, the pharmaceutical application of these flavonoids against different cancers using nanotechnologies such as polymeric, lipid-based nanoparticles (solid–lipid and liquid–lipid), liposomes, and metallic nanocarriers is addressed. Finally, combination therapies in which these flavonoids are employed along with other anti-cancer agents are described, indicating the effective therapies for the management of various malignancies.

## 1. Introduction

Cancer is a life-threatening disease, and is a current subject of choice on which biomedical scientists work with the aim of establishing safe and effective chemotherapy [1]. Cancer is one of the primary causes of death worldwide, with its metastasis leading to the migration of cancerous cells that could potentially affect multiple vital organs of the body [2]. To date, chemotherapy has been the primary clinical treatment, with associated complications that include drug resistance of the cancerous cells and off-target side effects, with an additional limitation being the cost-effectiveness of the therapies [3]. Furthermore, complications arising from this treatment option could be fatal for the older age group of patients (geriatric). In this context, there are many other potential treatments that are able to reduce the lethality of disease by adjuvating the primary chemotherapy treatment [4,5].

In order to uncover various possibilities, researchers have explored the anticancer potential of natural compounds, and in recent decades, there has been an increased interest in herbal drugs, especially in the context of underdeveloped nations, as a number of plant species have been demonstrated to possess exceptional anticancer effects [6]. Additionally, the introduction of phytochemicals as potential chemopreventive agents against t cancer is highly desirable due to their safety, low toxicity, and widespread acceptance as herbal remedies [7]. Therefore, in addition to synthetic chemotherapeutics, promising outcomes have already been achieved with natural products such as taxols, vincristine, and etoposide against a variety of cancers. Therefore, considering the success of therapies using natural compounds, scientists have explored more plant-derived compounds with respect to their action against specific cancers [8]. The recent work in the field is also focused on the development of delivery methods for phytochemicals, mainly with respect to polyphenols, which are currently being used to prevent and treat different types of cancer malignancy. Additionally, the different mechanisms of these compounds have been shown to possess promising anticancer effects with the synthetic anticancer agents, and many combinations have already been shown to exhibit synergistic chemotherapeutic effects by resensitizing cancer cells, thus reducing their resistance towards chemotherapy. For instance, many polyphenolic compounds, including quercetin and kaempferol, reduce the chemoresistance of cancer cells, making them more sensitive towards the active anticancer compound. Therefore, in light of abundant evidence, it can be said that natural compounds have the potential to support the primary chemotherapy treatment, as has been demonstrated in a range of in vitro and in vivo experiments [9]. In line with this, we explored and reviewed the flavonoid compounds that have shown significant chemotherapeutic effects in a range of different anticancer cells and in vivo models.

Flavonoids, the most common and widely distributed class of phytochemicals, are present in almost all plants. They are widely present in edible fruits, vegetables, and plant-derived products such as tea, cocoa and wine. Flavonoids can be categorized into a number of subgroups, such as flavanones, isoflavones, flavones, and flavanols. These compounds are polyphenolic, with their basic skeleton structure consisting of various hydroxyl functional groups. The skeleton of these compounds consists of two aromatic rings and a heterocyclic pyran ring. Based on the position of functional groups onto pyran ring, the antioxidant potential varies, accordingly imparting differing effects. These compounds have the potential to prevent cancer, reduce inflammation, and fight oxidative stress. In recent studies, the beneficial effects have not been limited to cancer malignancies, showing promising effects in other diseases, such as immune, cardiovascular and neurological diseases. [10]. Therefore, there is currently a huge market for these compounds, which are being used for supplementary and adjuvant therapy [11].

With respect to the potential anticancer properties of these compounds, a variety of preclinical investigations have demonstrated their effectiveness for the treatment of cancer. The main mechanism of such compounds is to decrease oxidative stress by scavenging Reactive Oxygen Species (ROS) and chelating metal ions. In addition, flavonoids inhibit the proliferation of cancer cells by downregulating the epidermal growth factor receptors (EGFR) and nuclear factor kappa B (NF-kB). Notably, the role in the inhibition of immune responses and COX enzymes significantly contributes to the anti-inflammatory effects. Moreover, these compounds exert direct effects by reducing ROS production, suppressing the Walburg effect, and inducing apoptosis, whereas indirect effects occur through stimulation of ATP production, preservation of membrane integrity, and total uncoupling of respiration, which can mainly be attributed to mitochondrial functions. These effects are responsible for the reduction in overall oxidative stress, which is considered to be a key factor in the induction of various malignancies.

To advance the potential of these compounds, bioavailability is crucial with respect to systemic circulation, as well as at the target site of the therapy. In this regard, nanotechnology has resulted in extraordinary outcomes by improving the solubility, permeation, and targeting ability of flavonoids at the target site. It has been demonstrated that a variety of delivery platforms, including liposomes, polymeric micelles, dendrimers, polymeric nanoparticles and microemulsions, are effective alternatives for the delivery of flavonoids to target tumor cells [12]. These nanoplatforms have been investigated for the last decade in terms of their ability to deliver such compounds, and their suitability for the effective delivery of flavonoids has been proven. In vitro and in vivo investigations against various cancer cells and animal models have recently validated and confirmed the application of these nanocarriers [3,13].

**Figure 1:**
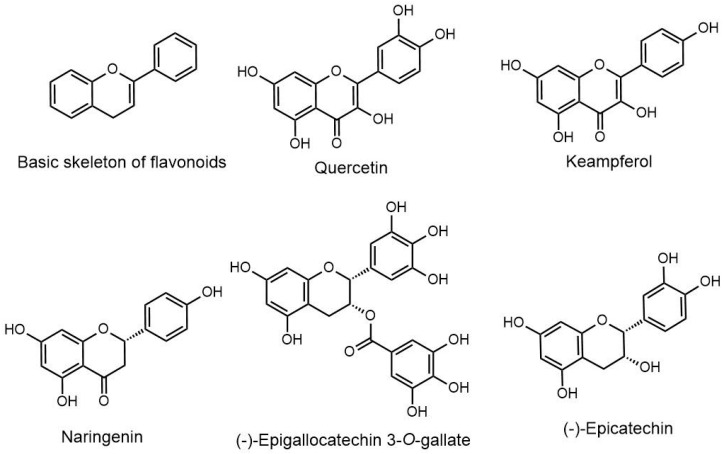
Chemical structures of the different flavonoids.

This review primarily focuses on the comprehensive anticancer effect of flavonoids such as naringenin, epicatechin, quercetin, epigallocatechin gallate and kaempferol (Figure 1) against various cancers, including pancreatic, prostate, breast, and colorectal cancers, as well as melanoma. The molecular mechanism of each compound in inhibiting the initiation of cancer is also covered. Subsequently, the application of nanotechnology for improved and effective delivery of such compounds is addressed in the manuscript. We also focus on the outcomes of a wide range of experimental and clinical studies on the anticancer activity of flavonoids in cancer cells and animal models. Finally, the studies reporting a combination of flavonoids and synthetic anticancer agents and their outcomes are included in this article and broadly reviewed.

## 2. Molecular Mechanism of Flavonoids in Different Type of Cancers

The molecular mechanisms of different flavonoids (quercetin, kaempferol, epigallocatechin gallate, epicatechin, and naringenin) against various cancers are described in the following section.

### 2.1. Epicatechin

Epicatechin (EC) is a well-known flavanol that is present in tea, cocoa, vegetables, fruits, and grains, and which protects human health by acting as an antioxidant and anti-inflammatory, improving muscle performance, lowering the symptoms of cardiovascular and cerebrovascular illnesses, and preventing diabetes and neurological disorders. EC has been reported to possess anticancer properties against various types of cancer. In breast cancer, EC reduces cell viability and induces apoptosis through mitochondrial intrinsic pathway. Induction of apoptosis has been found to be associated with elevated ROS production and subsequent downstream pathways [14]. In breast cancer, epicatechin (200 nM) mimics the effects of 100 nM testosterone in inducing apoptosis in PC3 cells. Recently, it was reported that epicatechin interacts with membrane androgen receptor ZIP9 as an agonist, subsequently mediating androgen-induced apoptosis in prostate (PC-3 cells) and breast cancer (MDA-MB-468 cells) cell lines [15]. A study conducted by researchers investigating lung cancer showed that EC enhanced the sensitivity of curcumin by increasing the serum level of curcumin, reducing cell growth and increasing apoptosis by enhancing GADD153 and GADD45 gene expression, which is associated with the MAPK pathway [16]. This increases the activity of mitochondria and oxygen consumption in Panc-1 cells, which are a type of pancreatic cancer cell, while having no effect on human normal fibroblasts. When used in combination with radiation therapy, epicatechin was also shown to increase the sensitivity of Panc-1, U87, and MIA PaCa-2 cancer cells to radiation, enhancing the activation of certain cellular signaling pathways involved in cell cycle regulation, including Chk2 phosphorylation and p21 induction, albeit only in cancer cells, and not in normal cells [17].

### 2.2. Epicatechin Gallate

Epicatechin gallate (EGCG) is a polyphenol found in green tea that is a natural antioxidant, possessing the highest antioxidant and free-radical-scavenging properties [18]. EGCG inhibits lipid peroxidation, suppresses ethanol-induced CYP2E1 upregulation (which produces ROS), and minimizes the formation of free radical protein adducts. It has been reported to exhibit anticancer properties in vivo and to impede the growth of a range of cancers in vitro [19]. By regulating intracellular signaling pathways, ECG or EGCG might interact with several molecules, including proteins, transcription factors, and enzymes, to suppress various phases of carcinogenesis [20]. EGCG has been reported to inhibit the initial carcinogenesis process in different types of cancers. Additionally, inhibition of cell proliferation, metastasis, angiogenesis, and induction of apoptosis have been the established mechanisms by which EGCG acts against cancer [21]. In addition, this compound regulates a number of signaling pathways, such as PI3K/Akt/mTOR, NF-κB, and Wnt/β-catenin, ultimately suppressing the growth of cancer cells [22].

### 2.3. Kaempferol

**Figure 2:**
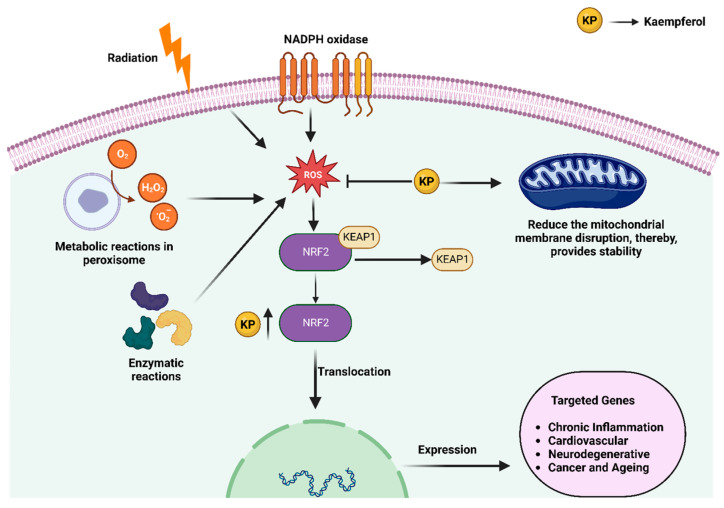
Antioxidant mechanisms of action of kaempferol [13]. Created using BioRender.com (accessed on 10 April 2023).

Kaempferol is present in a range of common vegetables and fruits. It has been shown to possess anti-inflammatory potential, effectively reducing the occurrence of the initial stages of disease development [23]. Figure 2 illustrates the antioxidant potential mechanism of kaempferol. It plays a protective role against various cancers, including lung, breast, colon, and pancreatic cancer, through the initiation of apoptosis and cell cycle arrest. In lung cancer, kaempferol decreases cell viability, reduces colony formation, and increases apoptosis [24,25,26]. Sanoki et al. reported the anticancer role of kaempferol in A549 lung cancer cells by reducing claudin 2 expression through inhibition of transcription factor STAT3 binding in the promotor region [24]. At the same time, Nguyen et al. highlighted the anticancer activity of kaempferol in lung cancer cells through activation of MAPK, decreasing Akt-1 activation-mediated Caspase 7 and PARP cleavage [25]. A study by Kuo et al. reported that kaempferol sensitizes lung cancer for radiotherapy by downregulating ERK and PI3K phosphorylation in vitro and in vivo. Kaempferol treatment in A549 cells induces G2/M-phase cell cycle arrest and reduces colony formation, while kaempferol in A549 tumor-bearing BALB/c mice enhances radiotherapy-induced tumor killing. Kaempferol-3-O-rutinoside, derived from *Tetrastigma hemsleyanum*, suppresses lung cancer by regulating calcium signaling and effectively triggering cytoskeleton collapse, mitochondrial dysfunction, and consequent calcium overload in order to achieve apoptosis [26]. Similarly, in HePG2 liver cancer cells, kaempferol decreases the proliferation, migration, and invasion by downregulating miR-21, upregulating PTEN, and inactivating the PI3K/AKT/mTOR signaling pathway [27]. Furthermore, in cholangiocarcinoma, kaempferol decreases Bcl-2 and increases levels of Bx, Fas, cleaved Caspase-3, 8, 9, and PARP [13,28].

**Figure 3:**
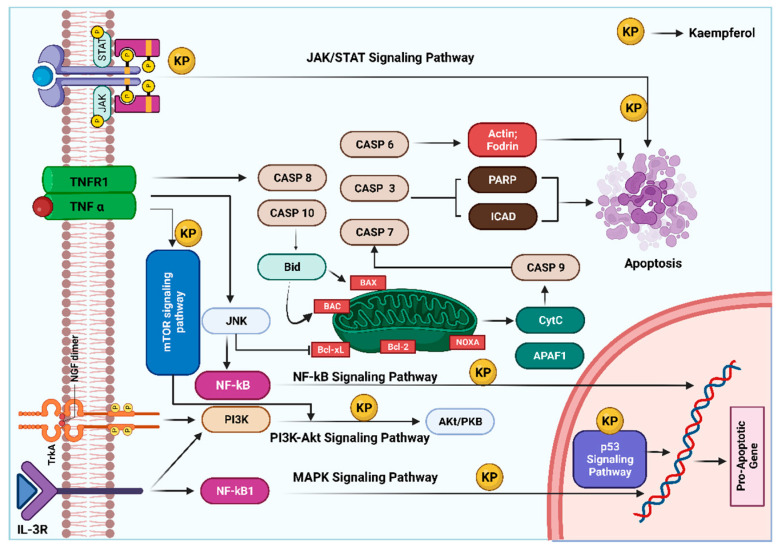
Anticancer role of kaempferol: at a molecular level, this compound is able to exert anti-cancer effects primarily by reducing the levels of proteins involved in the development and progression of cancer. This is accompanied by induction of apoptosis, arrest of the cell cycle, and downregulation of anti-inflammatory proteins. In addition, different molecular signaling pathways and mitochondrial mechanisms are involved in the activation of tumor cell apoptosis by kaempferol. The illustration is adopted with modifications from Amjad et al. [30]. Created with Biorender.com (accessed on 10 April 2023).

In addition, AKT, TIMP2, and MMP2 have been shown to be downregulated by kaempferol treatment, decreasing Ki-67-positive cells in vivo, resulting in decreased proliferation and invasion [28]. In skin cancer, kaempferol binds to the ATP binding pocket of RSK2 and MSK1, inhibits their kinase activities, and reduces solar UV-induced CREB and histone H3 phosphorylation [29]. RSK2 and MSK are potent kinases that possess key roles in the neoplastic transformation of human skin cells induced by solar UV radiation [29]. The anti-cancer role of kaempferol is presented in Figure 3.

### 2.4. Naringenin

Naringenin is one of the main flavonoids, and is mainly found in fruits such as citrus and figs. This compound demonstrates a range of therapeutic potentials in terms of reducing lipid peroxidation and increasing antioxidant defense by scavenging free radicals [31]. It prevents the occurrence of fatty liver complications by modulating the pathways associated with fatty acid metabolism. Additionally, promising outcomes have been obtained in the management of various cancers, including skin cancer, breast cancer, lung cancer, and the management of solid tumors [32]. Various in vitro studies have demonstrated its effectiveness as an approach to combatting these malignancies. The anticancer and antiproliferative potential of naringenin is associated with the inhibition of signaling pathways, and significant results have been reported through repairing DNA. The anti-tumor efficacy of this compound exhibits a dose-dependent therapeutic efficacy. The antioxidant mechanism of naringenin acts distinctly for each type of cancer [33,34]. For example, it demonstrates antiproliferative activity, decreasing cell proportion at a specific cell cycle phase (Sub G0/G1) for the management of melanoma [35]. In the case of breast cancer, it acts as a proapoptotic and anticancer compound by inhibiting HER2-TK activity. Additionally, it inhibits breast cancer by decreasing the secretion and disposition of TGF-β1 [36]. Its antiproliferative, proapoptotic, and anti-tumor activity has been evaluated and investigated in a range of cancer cells [37]. Hence, it can be said that this compound demonstrates an effective and promising therapeutic potential for the prevention and treatment of different types of cancer.

### 2.5. Quercetin

Quercetin is another important flavonoid that is present in various fruits and vegetables and can be readily accessed through food diets [38]. Various in vitro and in vivo studies have revealed quercetin’s anticancer therapeutic potential in different cancer types [39,40]. It exhibits anti-tumor activity by hampering the progression of the cell cycle, downregulating cell proliferation, angiogenesis, and metastasis; furthermore, it has been reported for the promotion of apoptosis [41,42]. However, the mechanism of each pathway for combating cancer is distinct. It causes cell cycle arrest through downregulating the cyclin D and cyclin E and upregulation of cyclin B [42,43]. Several studies have validated the potential of quercetin on the basis of in vitro and in vivo investigations. Hashemzaei et al. explored this compound by performing an MTT assay, and the results indicated that a significant effect was exerted by quercetin through induction of apoptosis in an in vitro model [44]. The outcomes of testing in animal models were significantly positive, with quercetin successfully reducing the tumor volume in animals.

**Figure 4:**
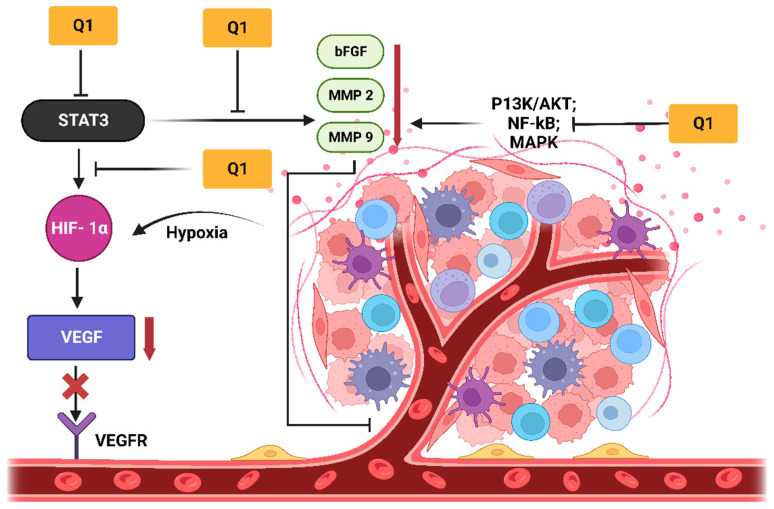
Anti-angiogenesis effects: quercetin (Q1) significantly inhibits the activation of VEGF by downregulating signaling molecules such as AKT and NF-kB, demonstrating the direct inhibition of VEGFR on the surface of endothelial cells. Additionally, the inhibition of the JAK- STAT3 pathway exerted anti-inflammatory and antitumor activities through the downregulation of MMP 2, MMP 9, TNF-α and bFGF. The inhibition of these factors retarded angiogenesis. VEGF and bFGF are two crucial factors in angiogenesis that upregulate the proliferation, migration, and formation of endothelial cells. The diagram was adopted with modifications from Asma et al. [30]. Created with Biorender.com (accessed on 10 April 2023).

Similarly, a report in Nature demonstrated the anticancer effects of quercetin in distinct cell lines. The findings revealed that quercetin induced cytotoxic effects in human leukemia and breast cancer cells in a dose-dependent manner. The investigation, performed by means of flow cytometry, indicated that quercetin caused S-phase arrest. The overall results indicated the existence of an intrinsic pathway by directly interacting with DNA, which could be a mechanism for the induction of apoptosis [45]. An overview of the anticancer potential and anti-angiogenesis of quercetin is presented in Figure 4.

## 3. Pharmaceutical Application of Flavonoids against Various Cancers Using Nanotechnological Approaches

The concept of the application of nanotechnology in disease treatment was proposed by Siddiqui and associates [46]. The utilization of nano-delivery systems to achieve cancer targeting has been proven to be an effective approach for obtaining better therapeutic outcomes [47,48,49]. As nanoparticles are able to bypass the natural barriers in the body, they can achieve effective release kinetics, and can easily target the desired locations [50,51]. Various studies have reported that flavonoids show low solubility, poor absorption and rapid metabolism [52]. In addition, food and other dietary compounds, when given in combination with flavonoids, tend to form complexes, precipitating the active moiety. As a consequence, pharmacokinetic biodistribution of flavonoids is not achieved [10]. Hence, there is a need to overcome these limitations by approaching nanotechnology and improving the anticancer activity of flavonoids by designing it in nano-engineered particulate drug delivery systems [53,54]. The key application of nanomedicine in cancer targeting is aimed at determining the therapeutic index (TI), which represents the overall bioavailability of the formulation [10,55,56].

### 3.1. Epigallocatechin-3-Gallate (EGCG)

EGCG has been proven to inhibit tumor cell growth in prostate, liver, and breast cancer cells when delivered in a nanoparticle formulation. The encapsulation of EGCG in polyethylene glycol polymer resulted in a 10-fold increase in anticancer activity [52]. The use of the conjugation technique to develop gold nanoparticles of EGCG has been demonstrated to offer significant chemotherapeutic effects. The main advantage of these nanoparticles is that they are smaller in size, less toxic, and non-immunogenic [51]. Another study by Rocha reported that the bioavailability of EGCG can be increased along with antioxidant properties by integrating EGCG in the polymeric carbohydrate complex. The use of carbohydrates as a matrix material stimulates apoptosis in Du145 prostate cancer cells [57].

Additionally, Liao et al. reported that docetaxel nanoparticles, when encapsulated in EGCG-nanoethosomes, reduce the lump size of cancer cells and boost anticancer activity [58]. EGCG plays a vital role in skin cancer treatment by initiating the antioxidant cycle in cells, ultimately leading to a decline in the reactive oxygen species (ROS) and enhancing anticancer activity [59]. It has been reported that green tea contains polyphenol groups responsible for reducing carcinogenesis. EGCG is one of the crucial polyphenols, and has been demonstrated to increase apoptosis and inhibit cell growth in skin cancer [60].

### 3.2. Quercetin

In vitro cell line studies have proved that quercetin inhibits cell cycle and cell proliferation [39]. On the other hand, in vivo studies of quercetin-loaded nanoparticulate systems have demonstrated an anti-cancer effect against the A549 pulmonary cell line [61]. Mandal et al. reported that quercetin inhibits hepatocellular carcinoma when encapsulated in PLA nanoparticles [62]. According to reports, quercetin circulates longer in the bloodstream when formulated using PEG nanoparticles [63]. Dora et al. concluded that the formulation of a PEG emulsion of quercetin could be used to enhance cytotoxic activity against the melanoma cell line B16F10 [64]. PLA–gold nanoparticles containing quercetin have been shown to block the cyto-c pathway in liver cancer cells [65]. This acts on the p53 protein, which changes the target site in carcinogenesis, in turn enhancing apoptosis, while caspase 3, 9 activity leads to tumor reduction and fatality in skin cancer cells [39]. Recent studies have proven that using lipids as the base complex of the formulation results in greater cytotoxicity than in the case of the normal suspension formulation of quercetin. The formulation of nanomicelles of quercetin and PEG–lipid as a complex can improve the anticancer activity in lung cancer cells [66]. In addition, magnetic nanoparticles are potential candidates for tumor targeting and for the inhibition of cell proliferation in different cancer cell lines (MCF-7, HePG-2, and A459) [67].

### 3.3. Naringenin

Recent studies have demonstrated that nanoparticle formulation of naringenin can restrain carcinogenesis in oral, lung, and colon cancer [68]. Notably, naringenin blocks the kinase pathway, ultimately leading to the inhibition of growth and, finally, the death of cancer cells [68]. Naringenin nanoparticles with chitosan as an encapsulating agent have been shown to exert an anti-cancer effect on lung cancer cell lines (A549) [69]. Fuster et al. documented that when formulated with silk fibroin, naringenin demonstrates cytotoxic effects on cervical cancer [70]. Naringenin nanoparticles, when designed with polycaprolactone, have been proven to demonstrate effective cytotoxic activity against lung cancer. Parashar et al. reported in vivo studies showcasing that drug targeting could be effectively achieved for lung cancer treatment in a rat model [71]. Naringenin has been effectively proven to exert therapeutic, anticancer activity in skin cancer cell lines (A431) [72].

### 3.4. Kaempferol

Kaempferol is prominently present in the range of fruits and plants such as red berries and citrus fruit. It blocks the enzymatic pathway phosphatidylinositol-3-kinase, which is responsible for cell proliferation and apoptosis [73]. It has shown anticancer effects in ovarian cancer cells by blocking carcinogenesis in OVCAR-3 [74]. A cytotoxic effect was observed that was related to the concentration of drug, whereby the viability of ovarian cancer cells decreasing with the use of PLGA as a polymeric nanomaterial [74]. Kaempferol-loaded chitosan NPs have demonstrated inhibitory effects on glioma cells in an animal model [75]. Saravanan et al. reported the development of gold nanoclusters of kaempferol, which were shown to be cytotoxic against A549 lung cancer cell line by inhibiting cell proliferation [76]. In another study, Chao et al. developed a transdermal delivery system for kaempferol consisting of submicron emulsion, and elucidated the fact that suitable systems can control the flux in skin and drug deposition of any drug, resulting in higher uptake in cancer models [77]. It was shown to possess anticancer activity against melanoma cells via an anti-proliferative mechanism and the promotion of apoptosis [78].

### 3.5. Epicatechin

High levels of epicatechin is present in green tea, providing antioxidant properties. Epicatechin possesses a low therapeutic effect due to its high affinity towards oxygen and light. Reports have described that encapsulation of the drug in nanoparticles can enhance its solubility, resulting in flavonoids’ exerting a better cytotoxic effect [79]. Chitosan, when used in the nanoparticle development of epicatechin, illustrates superior anticancer activity against breast cancer cells by providing sustained release of drug [80]. Epicatechin causes the denaturation of DNA, causing apoptosis in myeloid cells of rats by nano-formulation. It blocks the interchange of Na^+^/H^+^ ions through cancer cells, ultimately leading to an imbalance in fluidity and pH [81]. Ravindranath et al. suggested that ingestion of epicatechin could help to avoid the spread of gastric cancer [82].

## 4. Combinations of Flavonoids with Synthetic Anticancer Agents

The use of flavonoids alone or in combination with nano-formulations has shown promise in terms of enhancing their therapeutic potential, bioavailability, and safety profile. With cancer being a major threat to public health, combination treatment using flavonoids by means of innovative drug delivery systems could be a potential therapeutic strategy. Several studies have demonstrated the significant potential of flavonoids in cancer therapy. In in vitro assays, flavonoids have shown significant cytotoxic effects against cancer cells. For example, quercetin in combination with catechin was able to inhibit breast cancer cell proliferation and cell cycle progression [83]. In one of the studies, human breast cancer cells (PMC42) and gut (HuTu-80 and Caco-2) cells were used to illustrate the synergistic effects of quercetin and kaempferol in the inhibition of cancer cell metastasis [84]. Aglycone flavonoids, such as quercetin, kaempferol, and naringenin, when combined, inhibited cancer cell proliferation in the Hepa-1c1c7 mouse liver cancer cell line and the LNCaP human prostate cancer cell line in a dose-dependent manner, with no cytotoxicity [85]. Ellagic acid and quercetin were able to be combined synergistically to elicit acute cell cycle arrest in human leukemia cells through the induction of apoptosis [86]. Additionally, ellagic acid significantly improved the efficacy of quercetin in decreasing viability and proliferation while inducing apoptosis (5 and 10 micro mol/L), respectively. Additionally, there were noticeable differences in cell cycle dynamics, with the interaction of ellagic acid and quercetin demonstrating the superior anticarcinogenic potential of polyphenolic combinations, focusing not only on the additive effect of individual components but also on synergistic biochemical interactions related to proliferation, cytotoxicity and apoptosis in MOLT-4 human leukemia cells [87]. The effects of quercetin, myricetin, and epicatechin were studied with respect to their impact on the development, morphology, and enzyme suppression of MCF7 human breast cancer cells [88].

When resveratrol at 50 µM was incorporated with quercetin at various doses (10, 25, and 50 µM), the inhibitory effects of quercetin on cell proliferation for oral cancer cells were significantly enhanced. It was concluded that amalgamated resveratrol and quercetin were potent growth and proliferation inhibitors of oral squamous carcinoma cells (SCC-25), thus necessitating their further investigation as cancer chemoprotective drugs [89]. Furthermore, combined therapy with resveratrol and quercetin prevents human leukemia cells from metastasizing. Similarly, the anti-tumor effect of the same combination on HT-29 colon cancer cells has also been investigated. It was observed that the combination of resveratrol and quercetin demonstrated greater anticancer properties and was able to suppress carcinogenic microRNA-27a [90]. Notably, the combination of quercetin and kaempferol was more effective at inducing cytotoxicity than either drug alone. In HCT-1 cells, the ratio (2:1) of combination of quercetin and kaempferol dose was tested. It exhibits decrease in proliferation and inhibition in cell growth in the G2/M phase [91]. Table 1 shows different combination systems of flavonoids with synthetic anticancer agents, whereas Table 2 demonstrates the findings of flavonoid–flavonoid combinations against various cancer cells.

### 4.1. Prostate Cancer

Prostate cancer is one of the leading causes of death, and, recently, a range of compounds have been tested for treatment of this malignancy. In a series of studies, the group of Kikuchi et al. demonstrated activation of the intrinsic apoptosis pathway, G1 phase arrest, and expression of PTEN, a significant negative regulator of the PI3K/Akt, and naringin, a polyphenolic flavonoid derived from grapefruit and other citrus fruits that has been proven to possess a chemo-sensitizing effect, synergistically strengthening the anticancer potential of paclitaxel in human prostate cancer cells, regardless of androgen dependence [115]. Furthermore, the PI3K/Akt axis was suppressed, and apoptosis was greatly increased when docetaxel and quercetin were coupled. Quercetin reversed the resistance to docetaxel in prostate cancer by activating the androgen receptor and the PI3K/Akt signaling pathways. Additionally, docetaxel-resistance-related qualities such mesenchymal and stem-like aspects, PI3K/Akt activation, and excessive P-glycoprotein expression may be reversible by employing quercetin [94]. Quercetin and 2-Methoxyestradiol increased antiproliferative and proapoptotic activity in both androgen-dependent LNCaP and androgen-independent PC-3 human prostate cancer cell lines, according to a study performed in vitro [94]. In an attempt to simulate the change in prostate cancer from AD (androgen dependency) to HRPC in an in vitro model, the effects of genistein, epigallocatechin gallate (EGCG) and quercetin were examined, all of which are abundant in traditional Asian diets [116].

### 4.2. Oral Cancer

Different flavonoids and synthetic anticancer compounds have been demonstrated to possess chemotherapeutic effects against oral cancer. In a study by Siddappa et al., the combination of curcumin and metformin was reported to have boosted the efficiency of chemotherapy against oral squamous carcinoma via a mechanism related to cancer stem cells [117]. Oral squamous cell carcinoma treatment resistance is significantly mediated by the p38 MAPK-Hsp27 axis. Quercetin and cisplatin can be used to target this axis in a bid to improve the prognosis of patients with oral squamous cell carcinoma [118].

### 4.3. Brain Cancer

Various combination chemotherapies have been demonstrated to exert significant therapeutic effects via a number of different mechanisms. Through the suppression of heat-shock protein 27, quercetin has been shown in vitro to sensitize human glioblastoma U87 and U251 cells, to temozolomide, an oral alkylating chemotherapeutic drug. In both in vitro and in vivo trials, the epigallocatechin gallate and anticancer combination therapies have demonstrated equivalent synergistic anti-tumor effects, with an average tumor volume reduction of about 70.3%. When considering research implying that EGCG therapy inhibits the ability of cancer stem cells to self-renew, this combination has shown a sizable effect [119]. A study by Martínez-Rodríguez et al. demonstrated that naringenin and low doses of cisplatin together increased the efficacy of the medicine by drastically limiting cell viability, enhancing the induction of cytotoxicity, and reducing the spheroid’s ability to invade the body [110].

### 4.4. Colorectal Cancer

One of the most widespread malignancies is colon cancer. Naringin and naringenin in combination have been demonstrated to be effective for overcoming multidrug resistance in carcinoma, which is a major obstacle to therapeutic treatment, and is brought on by a variety of defense mechanisms [35]. In drug-resistant colorectal cancer cells, kaempferol therapy drastically decreases glucose absorption and lactic acid formation. Via this the mechanism, kaempferol increases the expression of microRNA-326 (miR-326) in colon cancer cells. Taken together, the data suggest that kaempferol may be crucial in reducing the obstacles to 5-Florouracil treatment in cancer by modulating the miR-326-hnRNPA1/A2/PTBP1-PKM2 axis [101].

### 4.5. Breast Cancer

Recently Zhang et al. reported the potential of epigallocatechin gallate as a therapeutic supplement for the treatment of human metastatic breast cancer, providing evidence that it enhanced the effectiveness of radiotherapy in patients with breast cancer [120]. Additionally, it has been demonstrated that luteolin lowers nuclear factor erythroid 2-related factor 2 (Nrf2)-driven activation and prevents STAT3 from making human breast cancer MDA-MB-231 cells more vulnerable to the drugs doxorubicin and paclitaxel [115].

**Table 2:** Various reported flavonoid–flavonoid combinations against various cancer cells as well as in vivo models.

S. No	Flavonoid	Flavonoid	Cancer Type	Findings	Model	Cell Type	Reference
1	Quercetin, Naringenin	Kaempferol	Liver; prostate	Exhibited synergistic chemotherapeutic potential against two different cells.	In vitro	LNCaP; Hepa 1c1c-7	[85]
2	Quercetin	Kaempferol	Gut; breast	Exhibited synergistic effect against HuTu-80 and Caco-2.	In vitro	HuTu-80; Caco-2	[84]
3	Ellagic acid	Quercetin	Leukemia	Exhibited apoptosis and reduction of cell growth in human leukemia cells (MOLT-4).	In vitro	MOLT-4	[86]
4	Resveratrol	Quercetin	Colon	Enhanced chemotherapeutic potential was observed.	In vitro	HT-29	[90]
5	Resveratrol	Quercetin	Glioma	Induced senescence-like growth arrest in C6 rat glioma cells.	In vitro	C6	[121]
6	Quercetin	Catechin	Breast	Inhibited mammary tumor growth and metastasis in nude mice.	In vivo	MDA-MB-231	[122]
7	Kaempferol	Resveratrol	Prostate	Inhibited TNF-α and cytokine IL-10.	In vitro	RAW-264.7	[123]
8	Naringenin	Quercetin	Breast	Showed anticancer potential against MCF-7 breast cancer cells.	In vitro	MCF-7	[124]
9	Quercetin	ECGC	Prostate	Enhanced antiproliferative activity in androgen-independent PC-3 cells and in androgen-dependent LNCaP prostate cancer cells.	In vitro	PC-3; LNCaP	[125]
10	Quercetin	Catechin	Breast	Inhibited the primary tumor growth of breast cancer xenografts in a nude mouse model.	In vivo; in vitro	MDA-MB-231	[83]
11	Quercetin	Naringenin	Liver	Exhibited significant potential in reduction of carcinogenesis.	In vivo	-	[126]

## 5. Regulatory Prospects for Polyphenolic Compounds

Currently, these compounds have not been approved by any of the regulatory authorities, including the USFDA, TGA, and EMA. To be approved for cancer prevention and treatment, these compounds would need to undergo several stages of research, including in vitro, in vivo and clinical trials. The involvement of a large number of patients would provide a valuable and significant dataset for the consideration and evaluation of the potential of compounds of any type for application in any kind of treatment, including cancer. However, in vitro data obtained under rigorous conditions indicates the potential of these compounds in cancer prevention and treatment. In parallel, such data do not support or validate the potential of such compounds for use in the treatment of cancer, and they have not yet been approved by any of the regulatory authorities.

## 6. Future Directions

The application of flavonoids has been reported in the literature for the treatment of many diseases, including cancer, as a result of their exceptional free radical scavenging properties. The different functions exerted by flavonoids enable these molecules to act on various targeted proteins to downregulate disease progression. There are a number of aspects in the application and translation of flavonoids that still need to be explored and investigated. Nevertheless, the existing literature demonstrates the wonderful and exceptional therapeutic effects exerted by flavonoids in in vitro and in vivo models. However, their translation from bench to bedside is still an area that needs to be explored. Although these compounds are available in the market in the form of nutraceuticals, such as antioxidant capsules and sachets for different applications (such as to play immunomodulatory functions and to improve heart conditions), these compounds have still not been applied in cancer prevention and treatment. In addition, the regulatory aspect of herbal compounds in the translation to clinics is still a puzzle that needs to be solved. Therefore, considering all of the evidence presented in the literature, there is a dire need to examine the translation aspect of these compounds for the treatment of different diseases, including cancer.

## 7. Conclusions

Flavonoids are natural compounds that can be obtained from a range of plants, and are abundantly present in different foods and beverages. The antioxidant behavior of these compounds makes them potent molecules of interest for consideration as a chemo preventive and in the treatment of various malignancies. Several studies have already been performed demonstrating the significant role of flavonoids in reducing and minimizing cancer induction in in vitro and in vivo models. In addition, these compounds have shown significant therapeutic effects by reducing inflammation, upregulating the immune response, and rejuvenating the normal function of cells. Therefore, these compounds can serve as wonderful therapeutics in the development of chemoprevention therapy against various cancers.

## Figures and Tables

**Table 1:** Various reported combined systems incorporating flavonoid and synthetic anticancer agents. The literature has reported several pieces of evidence; however, we have summarized only the more recent instances of combinations of flavonoids with synthetic anticancer agents.

S. No	Flavonoid	Synthetic Anticancer Drug	Cancer Type	Result	Model	IC_50_ Value	Cell Type	Reference
1	Quercetin	5-Flurouracil	Esophageal	Inhibited the growth of EC9706 cells and induced higher apoptosis compared to 5-Flurouracil alone.	In vitro	QCN = 100 µM; 5-FU= 0.2 mM	EC9706; Eca109	[92]
2	Quercetin	5-Flurouracil	Colorectal	Combination of quercetin and 5-Flurouracil reduced the growth of HT29 cells significantly compared to quercetin alone.	In vitro	QCN = 176.6 µg/mL; 5-FU = 107 µg/mL	HT-29	[93]
3	Quercetin	Docetaxel	Prostate	Quercetin in combination with docetaxel reversed drug resistance via P13K/AkT signaling pathways.	In vitro	QCN = 20 µM; DTX = 5 nM	LNCaP/R; PC-3/R	[94]
4	Quercetin	Docetaxel	Breast	These two drugs in combination provided synergistic effects and resensitized the cancer cells to cancer treatment.	In vitro	QCN = 64.8 µM; DTX = 5 nM	MCF-7	[95]
5	Quercetin	Docetaxel	Hepatic	Demonstrated superior anticancer efficacy with accumulation in tumor cells.	In vitro; In vivo	DTX-QCN = 0.00639 µg/mL	HepG2	[96]
6	Quercetin	Vincristine	Breast	Provided synergistic anticancer effects by delivery of both compounds to the cancer cells.	In vitro	-	MCF-7	[97]
7	Quercetin	Gemcitabine	Pancreatic	Demonstrated enhanced cellular uptake and improved cytotoxicity towards cancer cells. Interestingly, in combination, these drugs showed better therapeutic effects.	In vitro	GMC = 0.97 µM; QCN = 97µM	Mia-PaCa-2; PANC-1	[98]
8	Quercetin	Methotrexate	Osteosarcoma	Quercetin increased methotrexate cytotoxicity in cancer cells.	In vitro	QCN = 142.3 µM; MTX = 13.7 ng/mL	Saos-2	[99]
9	Kaempferol	5-Flurouracil	Colon	Showed synergistic inhibitory effects with respect to cell cytotoxicity. In addition, both drugs induced apoptosis and initiated cell cycle arrest. The blockade of ROS production by kaempferol and the modulation of various proteins validated the success of chemotherapy.	In vitro	KMP = 44 µM; 5FU = 26 µM	LS174	[100]
10	Kaempferol	5-Flurouracil	Colorectal	Kaempferol reversed 5-Fluorouracil resistance by downregulating PKM2-mediated glycolysis.	In vitro	KMP = 70µM; 5FU = 37 µM	LS174	[101]
11	Kaempferol	Cisplatin	Head and Neck Squamous	The combination was shown to inhibit the consumption of oxygen and metabolism, and reduced the ATP content in cancer cells.	In vitro	KMP = 120 µM; 40 µM	Cal-27; Hep-2	[102]
12	EGCG	Docetaxel	Prostate	EGCG in combination with docetaxel reduced the resistance of docetaxel towards cancer cells and increased the chemotherapeutic effects.	In vitro	EGCG = 40 µM; DTX = 5 nM	LAPC-4-AI; PA-3	[103]
13	EGCG	5-Flurouracil	Colorectal	EGCG was revealed to improve the sensitivity of colorectal cells for 5-Flurouracil by inhibiting and downregulating the GRP78/NF-kB/miR-155-p/MDR1 pathway.	In vitro	5FU = 5 µM; EGCG= 50 µM	HCT-116; DLD1	[104]
14	EGCG	5-Flurouracil	Oral Squamous cell	It was revealed that this combination significantly reduced both cell viability and cell migration compared to 5-Flurouracil alone.	In vitro	-	PE/CA-PJ15	[105]
15	EGCG	Doxorubicin	Pancreatic; Colon	This combination significantly induced apoptosis and blocked cell metastasis and progression by downregulating the ERK pathway.	In vitro	EGCG = 62 µM; DOX = 5 µM	Panc-1; MIA PaCa-2; BxPc-3; HCT15	[106]
16	EGCG	Gemcitabine	Pancreatic	EGCG with gemcitabine was revealed to downregulate the growth, invasion, and migration of cancer cells, causing apoptosis by hampering the STAT3 signaling pathway.	In vitro	EGCG = 60µM; GCM = 20 µM	AsPC-1; PANC-1	[107]
17	EGCG	Docetaxel	Prostate	The combination of these two reduced the tumor growth by 62 fold.	In vivo	-	CRPC	[108]
18	Naringenin	Paclitaxel	Prostate	It was revealed that naringenin sensitized the cancer cells for paclitaxel therapy by inducing apoptosis and cell cycle arrest in the G1 phase.	In vitro	NGN = 150 µM; PTX = 5 nM	DU145; PC3	[109]
19	Naringenin	Cisplatin	Cervical	It was revealed that naringenin impaired cell growth by initiating apoptosis, proliferation, and cytotoxicity.	In vitro	NGN = 500 µM; CSP = 16 µM	HeLa	[110]
20	Naringenin	Cisplatin	Lung	In combination with naringenin, the chemotherapeutic effects of cisplatin were significantly increased, with naringenin increasing the expression of caspase-3, and recuing the expression of MMP-2, and MMP-9.	In vitro	CSP = 28 µL/mL; NGN = 200 µM	A549	[34]
21	Epicatechin	5-Flurouracil	Gastric	In combination, epicatechin showed higher inhibitory effects on the production of lactate and exhibited higher cytotoxicity and ROS-mediated apoptosis in SNU620/FU cells.	In vitro	-	SNU620	[111]
22	Epicatechin	Docetaxel	Prostate/Breast	Higher chemotherapeutic effects were observed through the upregulation of CDKN1A, BAX, and caspase 9.	In vitro	-	PC3; DU-145; MCF-7	[112]
23	Epicatechin	Cisplatin	Lung	Epicatechin showed concentration-dependent cytotoxicity with cisplatin and promoted cell death by a exerting synergistic effect.	In vitro	-	A549/DDP	[113]
24	Epicatechin	Doxorubicin	Breast	In combination with doxorubicin, epicatechin reduced the chances of cardiotoxicity without altering the chemotherapeutic effects of doxorubicin in MDA-MB231 cells.	In vitro; In vivo	-	MCF-7; T47D; MDA-MB-231	[114]
